# High loading of trimethylglycine promotes aqueous solubility of poorly water-soluble cisplatin

**DOI:** 10.1038/s41598-021-89144-0

**Published:** 2021-05-07

**Authors:** Riki Kadokawa, Tetsuo Fujie, Gyanendra Sharma, Kojiro Ishibashi, Kazuaki Ninomiya, Kenji Takahashi, Eishu Hirata, Kosuke Kuroda

**Affiliations:** 1 Faculty of Biological Science and Technology, Institute of Science and Engineering, Kanazawa University, Kakuma-machi, Kanazawa, 920-1192 Japan; 2 Division of Tumor Cell Biology and Bioimaging, Cancer Research Institute of Kanazawa University, Kakuma-machi, Kanazawa, 920-1192 Japan; 3 Institute for Frontier Science Initiative, Kanazawa University, Kakuma-machi, Kanazawa, 920-1192 Japan; 4 Nano Life Science Institute of Kanazawa University, Kakuma-machi, Kanazawa, 920-1192 Japan

**Keywords:** Cancer, Drug discovery

## Abstract

Trimethylglycine (TMG) is a cheap, natural, and highly biocompatible compound. Therefore, it has been used in the fields of food and life sciences, but the application of solid TMG is limited to utilisation as an “additive”. In the present study, we focussed on the high solubility of TMG in water, derived from the aprotic zwitterionic structure, and proposed TMG as the chemical accounting for a major portion of the aqueous solution (e.g., 50 wt%). High loading of TMG shifted the properties of water and enabled the dissolution of poorly water-soluble cisplatin, an anticancer agent, at high concentration (solubility of cisplatin: 0.15 wt% in water *vs* 1.7 wt% in TMG aqueous solution). For hepatic arterial infusion, this can reduce the amount of cisplatin administered from 40 to 4 mL. It enables simple injection using a syringe, without the need for catheters and automatic pumps, leading to critical alleviation of the risk to patients. Furthermore, we produced a dry powder from a cisplatin-containing TMG aqueous solution via freeze-drying. Powders can be conveniently stored and transported. Furthermore, cisplatin is often used as a mixture with other drugs, and cisplatin aqueous solutions are not preferred as they dilute the other drugs.

## Introduction

Trimethylglycine (TMG) is present in various organisms and is especially rich in sugar beets. It is also the most commonly accumulated osmolyte in nature^1–3^. Therefore, TMG has been established as a low-price and highly biocompatible compound and used as an additive in food and cosmetic preparations. Furthermore, TMG has found application in life sciences as an additive, including as a polymerase chain reaction adjuvant^4^ and cryoprotective agent^5^. In other words, TMG has been employed as an additive to exert its functions in water as TMG is solid.

In the present study, we proposed the use of TMG to account for a major proportion of the aqueous solution (e.g., 50 wt%). TMG is a known natural aprotic zwitterion, presenting high water solubility owing to the charges, unlike most amino acids. High loading of TMG can drastically alter the nature of water, adding properties to water that cannot be realised by pure water or dilute aqueous solutions. Accordingly, the dissolution of poorly water-soluble drugs is a good candidate for these properties owing to the biocompatibility of TMG. Typically, the dissolution of poorly water-soluble drugs is achieved using organic solvents such as dimethyl sulfoxide (DMSO)^6–10^. However, organic solvents, including DMSO, are toxic and should be avoided in clinical and fundamental researches if possible^9,11–14^.

We employed a 50 wt% TMG aqueous solution (aq.) as a nearly saturated solution. In the present study, we used cisplatin as the poorly water-soluble platinum agent. Although cisplatin is a classical anticancer agent, it is used to treat certain cancer types such as triple-negative breast cancer. Dissolution of cisplatin in DMSO is especially avoided because chloride, as a ligand of cisplatin, is exchanged for DMSO, with the anticancer effect of cisplatin finally abolished^10,15^. Thus, we proposed a 50 wt% TMG aq. as an efficient cisplatin solvent without eliminating its anticancer effect.

## Results and discussion

Cisplatin is poorly water-soluble, with marginal solubility (approximately 0.15 wt%^16^). The 50 wt% TMG aq. dissolved 1.7 wt% of cisplatin (Table 1). The diluted TMG solutions (25 and 5 wt%) dissolved less than 1.0 wt% of cisplatin, indicating that high loading of TMG is required to functionalise and dissolve cisplatin. High loading of L-carnitine, an analogous natural aprotic zwitterion, with a solubility of approximately 70 wt% in water, functionalised water, and the concentrated L-carnitine aq. dissolved cisplatin (Table 1).

**Table 1:** Solubility of cisplatin in TMG or L-carnitine aqs.

	Content (wt%)	Solubility of cisplatin (wt%)
TMG	50	1.7
	25	< 1.0
	5	< 1.0
L-carnitine	70	1.4
	50	1.0
	25	< 1.0
	5	< 1.0
Water^a^	–	0.15^16^

^a^150 mM NaCl aq. at ambient temperature. The same solubility is shown in phosphate-buffered saline.

The 50 wt% TMG aq. dissolved cisplatin at an 11 times higher concentration than phosphate-buffered saline (PBS). Accordingly, the cisplatin solution administered to patients can be reduced to one-eleventh. For hepatic arterial infusion of cisplatin, 40–50 mL of a saturated cisplatin solution (0.15 wt%) is administered to a typical adult male using a catheter and an automatic pump. Theoretically, the highly concentrated cisplatin (1.7 wt%)/TMG solution reduces the amount to 4–5 mL, allowing injection using a syringe, without the need for catheters and automatic pumps, resulting in the critical alleviation of patient risk.

To discuss the dissolution mechanism of cisplatin in the TMG solution, the polarity of TMG was investigated. Ions have generally high polarity due to their positive- and negative charges. Since TMG does not have special functional groups, we considered the hydrogen bonding between TMG and cisplatin based on the high polarity should be a reason to solubilise. For solvents and organic ions, Kamlet-Taft parameters are generally used as indicators of the polarity^17,18^. In detail, *β* value in the parameters exhibits the hydrogen bond basicity and the calculated *β* value of TMG was 0.77. It is higher than that of water (around 0.15^17^), which does not dissolve cisplatin, and similar to that of cisplatin-dissolving DMSO (0.83^18^). Therefore, the hydrogen bonding between carboxylate anion of TMG and ammonia ligand is considered to be a key factor to dissolve.

**Figure 1:**
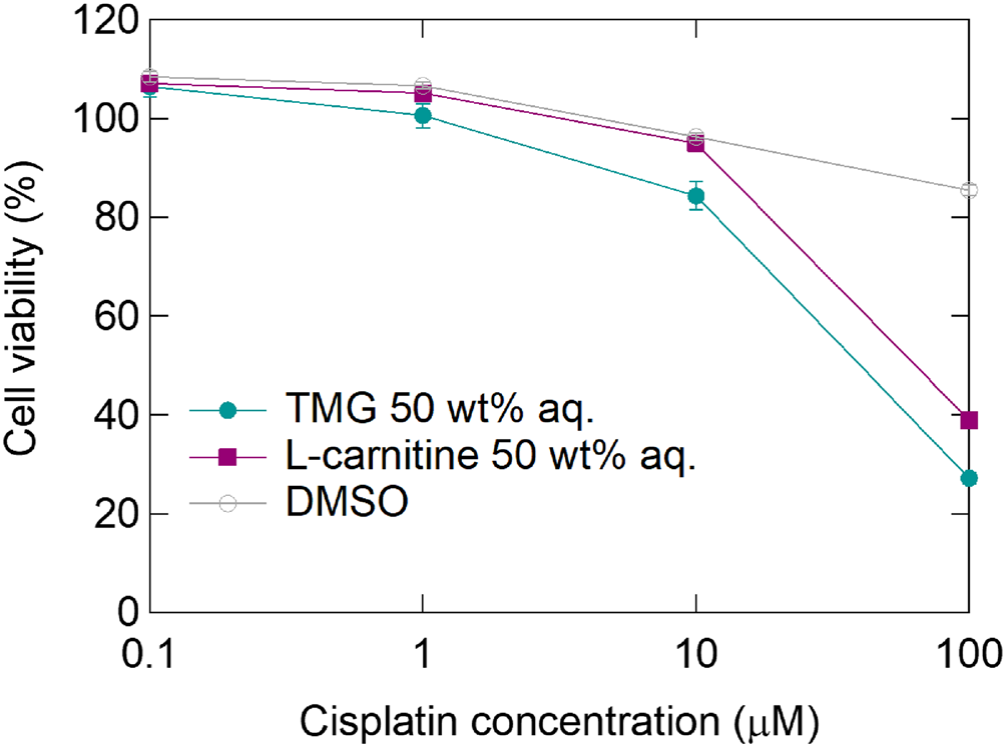
MDA-MB-231 cell viability after treatment with cisplatin dissolved in the indicated solvents. (n = 3, biologically triplicated).

We confirmed that cisplatin dissolved in the 50 wt% TMG aq. retained its anticancer effects. The 50 wt% TMG aq. containing 10 mM cisplatin was prepared and added to human breast cancer cells (MDA-MB-231) at final concentrations ranging between 0.1 and 100 µM, by dilution in a culture medium. After incubation for 72 h, cell viability was measured and was decreased in a dose-dependent manner (Fig. 1). On employing DMSO as the solvent, the anticancer effect of cisplatin was abolished, as previously reported^10^. Furthermore, cisplatin dissolved in a 50 wt% L-carnitine aq. also retained the anticancer effect.

**Figure 2:**
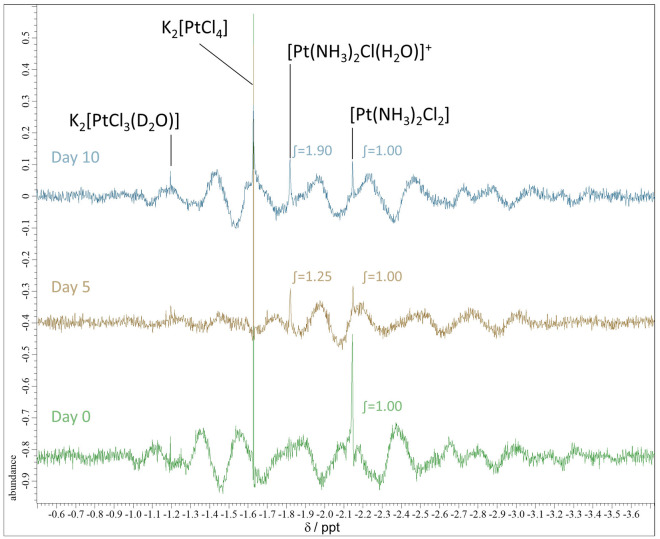
^195^Pt NMR spectra of cisplatin dissolved in 50 wt% TMG aq. Pt(NH_3_)_2_Cl_2_: − 2145 ppm, [Pt(NH_3_)_2_Cl(H_2_O)]^+^: − 1821 ppm. The x axis is shown in ppt. We used co-axial tubes and K_2_[PtCl_4_] in D_2_O was in the inner tube.

The ammonia ligand of cisplatin (Pt(NH_3_)_2_Cl_2_) is stable, but the chloride ligand is exchangeable in the TMG aq. The replacement species are presumed to be water- or carboxylate-containing TMG. Cisplatin in the 50 wt% TMG aq. was measured using ^195^Pt NMR spectroscopy (Fig. 2). K_2_[PtCl_4_] was used in H_2_O (− 1628 ppm; −1.628 ppt in Fig. 2)^19,20^ as a reference. A part of K_2_[PtCl_4_] was converted to K_2_[PtCl_3_(D_2_O)] in D_2_O, detected at − 1196 ppm. Immediately after dissolution, Pt(NH_3_)_2_Cl_2_ was observed at − 2145 ppm^21^ in the 50 wt% TMG aq. The absence of other peaks suggests that ligand exchange did not occur. The solution was left at room temperature and evaluated on days 5 and 10. Although Pt(NH_3_)_2_Cl_2_ was observed, another peak was observed at − 1821 ppm, indicating that the chloride ligand was exchanged. When the integral value of the signal of Pt(NH_3_)_2_Cl_2_ was fixed as 1.0, that of the emerging compound was 1.25 and 1.90 at days 5 and 10, respectively. The exchange proceeded over time, with 66 mol% of the chloride ligand exchanged after 10 days.

The emerging signal at − 1821 ppm can be derived from either of the following compounds: [Pt(NH_3_)_2_Cl(TMG)]^+^, [Pt(NH_3_)_2_(TMG)_2_]^2+^, [Pt(NH_3_)_2_Cl(H_2_O)]^+^, and [Pt(NH_3_)_2_(H_2_O)_2_]^2+^. Herein, we performed mass spectrometry for the cisplatin-dissolved TMG aq.; however, none of them were detected owing to the extremely strong signal of excess TMG. Therefore, we measured cisplatin dissolved in water. Despite the low solubility (0.15 wt%), it is sufficient for performing mass spectrometry. A signal for [Pt(NH_3_)_2_Cl(H_2_O)]^+^ (m/z = 282) and a slight signal for [Pt(NH_3_)_2_(H_2_O)_2_]^2+^ (m/z = 133) were detected (Fig. S1). Then, the same sample was subjected to ^195^Pt NMR spectroscopy (370,000 scans due to low solubility). A signal appeared at − 1839 ppm, in addition to the signal for Pt(NH_3_)_2_Cl_2_ (− 2148 ppm) (Fig.  S2). The new signal was assigned to [Pt(NH_3_)_2_Cl(H_2_O)]^+^, in accordance with the previous literature stating that signals for [Pt(NH_3_)_2_Cl(H_2_O)]^+^ and [Pt(NH_3_)_2_(H_2_O)_2_]^2+^ in water appear at approximately − 1841^21,22^ and − 1567 ppm^22,23^, respectively. [Pt(NH_3_)_2_(H_2_O)_2_]^2+^ observed by mass spectrometry was not detected by ^195^Pt NMR, presumably because of detection sensitivities and the low concentration of [Pt(NH_3_)_2_(H_2_O)_2_]^2+^.From these results, It was confirmed that chloride in cisplatin was exchanged not for TMG but for water in the 50 wt% TMG aq. after several days.

[Pt(NH_3_)_2_Cl(H_2_O)]^+^ should scarcely enter cells owing to their positive charges^24^. Conversely, the anticancer effect was maintained in the 50 wt% TMG aq., as shown in Fig. 1. The ligand exchange between Cl^–^ and H_2_O is known to be an equilibrium reaction^25,26^, and Cl^−^-rich solutions can cause ligand re-exchange from [Pt(NH_3_)_2_Cl(H_2_O)]^+^ to Pt(NH_3_)_2_Cl_2_. In the experiment shown in Fig. 1, the initial stock solution was prepared as 10 mM cisplatin dissolved in 50 wt% TMG aq., and then it was diluted to the desired concentrations with the medium before treating MDA-MB-231 cells. Hence, NaCl contained in the medium is assumed to induce ligand re-exchange even in the presence of TMG.

**Figure 3:**
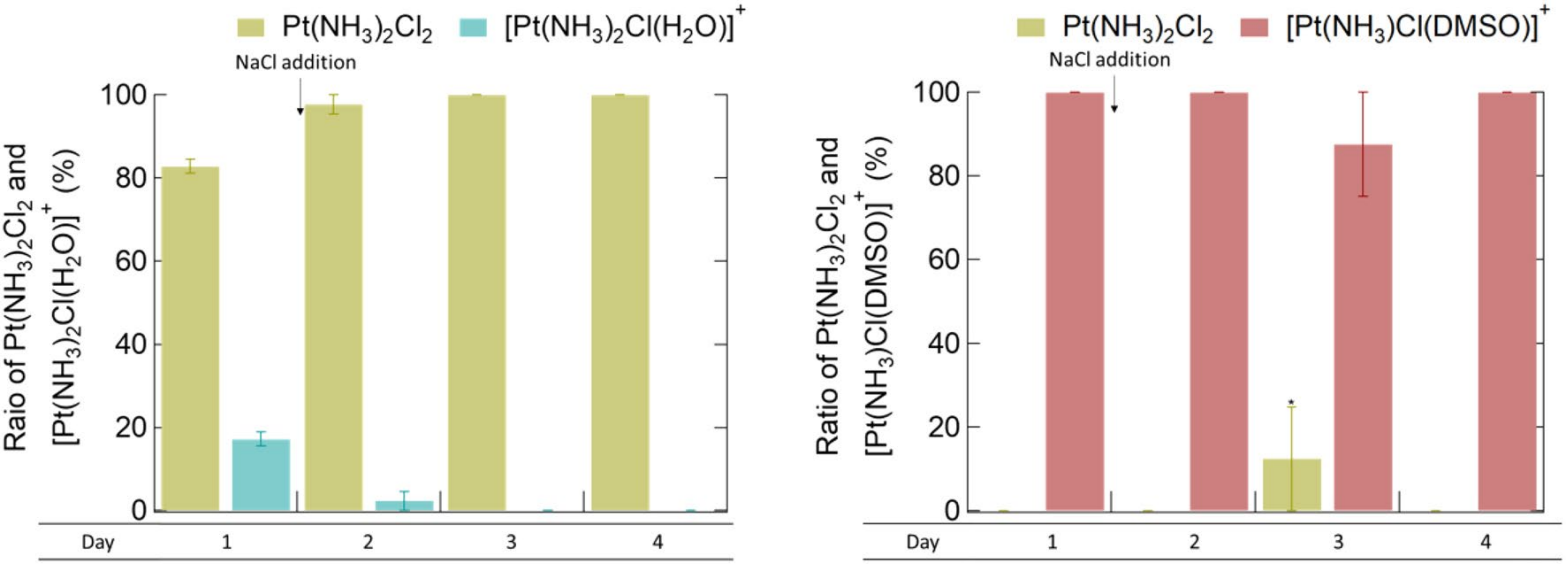
Ratio of Pt(NH_3_)_2_Cl_2_ and the substituted compounds after dissolution in 50 wt% TMG aq. or equimolar DMSO aq., followed by NaCl addition. *The peak of Pt(NH_3_)_2_Cl_2_ on day 3 in DMSO aq. was observed in only one of the three samples.

Cisplatin was dissolved in 50 wt% TMG aq., and then left for 24 h. The ratios of Pt(NH_3_)_2_Cl_2_ and [Pt(NH_3_)_2_Cl(H_2_O)]^+^ were 83 and 17 mol%, respectively, as measured by ^195^Pt NMR (Fig. 3, day 1). Later, NaCl was additionally dissolved, and the ratio was evaluated on days 2–4. As expected, [Pt(NH_3_)_2_Cl(H_2_O)]^+^ decreased: the ratios at days 2, 3, and 4 were 2, 0, and 0 mol%, respectively. Therefore, [Pt(NH_3_)_2_Cl(H_2_O)]^+^ reverted to Pt(NH_3_)_2_Cl_2_ in the 50 wt% TMG aq. in the presence of NaCl.

For comparison, the same experiments were performed using a DMSO aq. Pt(NH_3_)_2_Cl_2_ and [Pt(NH_3_)_2_Cl(H_2_O)]^+^ were not detected, and only [Pt(NH_3_)Cl(DMSO)]^+^ (− 3160 ppm)^15^ was detected on day 1. This trend was retained even after the addition of NaCl. A small amount of Pt(NH_3_)_2_Cl_2_ (− 2115 ppm) was observed on day 3. This indicated that [Pt(NH_3_)Cl(DMSO)]^+^ was possible to revert to Pt(NH_3_)_2_Cl_2_, but Pt(NH_3_)_2_Cl_2_ was detected only once during experiments performed in triplicate. It was confirmed that [Pt(NH_3_)Cl(DMSO)]^+^ in the DMSO aq. scarcely reverted to Pt(NH_3_)_2_Cl_2_ in the presence of NaCl, unlike [Pt(NH_3_)_2_Cl(H_2_O)]^+^. Based on these results, although ligand exchange of Pt(NH_3_)_2_Cl_2_ occurred in both the TMG aq. and DMSO (aq.), the replacing ligand species differed, which resulted in varying anticancer activities that were retained in the TMG aq. but not in DMSO.

**Figure 4:**
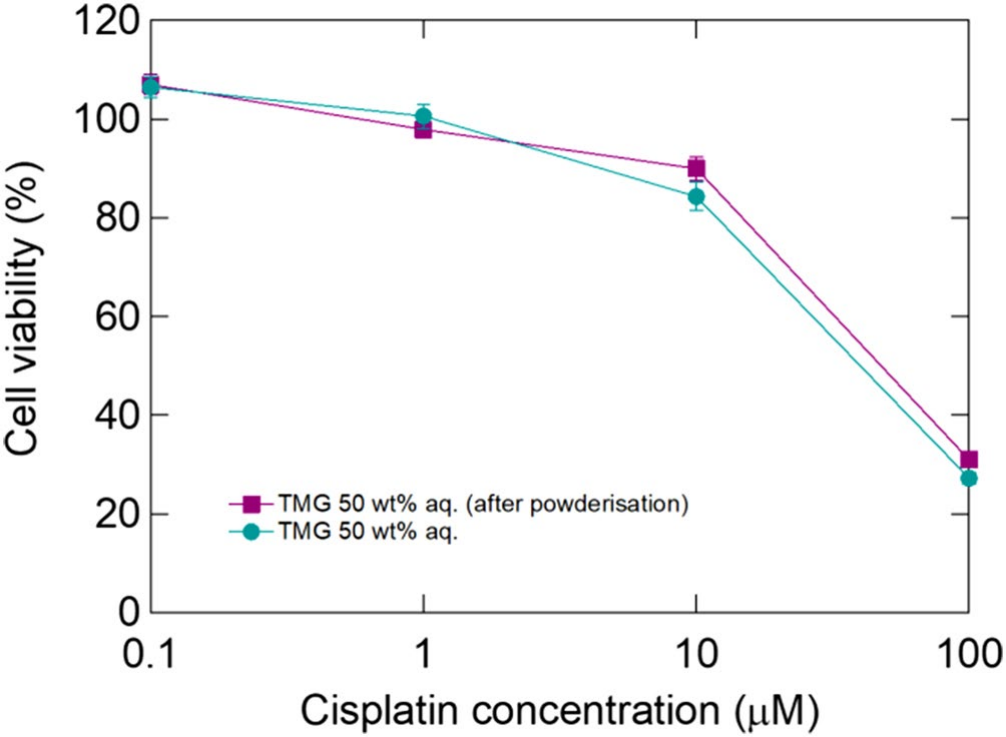
MDA-MB-231 cell viability after treatment with cisplatin dissolved in 50 wt% TMG aq., with or without powderisation. (n = 3, biologically triplicated).

The 50 wt% TMG aq. dissolved cisplatin at high concentrations and the resulting solution retained the anticancer effect of cisplatin. Herein, we proposed powderisation, as powders can be conveniently stored and transported. Furthermore, cisplatin is often used as a mixture with other drugs, and cisplatin aqueous solutions are undesirable as they dilute the other drugs. The 50 wt% TMG aq. containing 10 mM cisplatin was freeze-dried. The obtained powder was pale yellow, based on the colour of cisplatin. We regenerated 10 mM cisplatin-containing 50 wt% TMG aq. by adding the same amount of water to the powder to determine the anticancer effect. Then, the regenerated solution was added to MDA-MB-231 cells at final concentrations ranging between 0.1 and 100 µM. The regenerated cisplatin solution killed MDA-MB-231 cells in a dose-dependent manner (Fig. 4), indicating that freeze-drying did not affect the anticancer effect.

On dissolving the cisplatin/TMG powder, the powder was rapidly solubilised in water (Fig. S3). It requires only 60 s for dissolution whereas the typical dissolution of native cisplatin powder in 50 wt% TMG aq. required approximately 1 h. Therefore, the freeze-dried cisplatin/TMG powder is ready-to-use. Freeze-drying is considered an effective method for preparing rapidly soluble agents because resulting materials often have different crystallinities^27^. This could be a critical factor for rapid dissolution and TMG additionally might interfere the crystallisation of cisplatin. For hepatic arterial infusion, cisplatin-saturated PBS (0.15 wt%) is considered the state-of-the-art solution and needs to be heated at 50 °C to dissolve cisplatin. Conversely, the mixed powder was rapidly soluble at room temperature and could prepare an 11-fold concentrated cisplatin solution; the cisplatin/TMG powder is extremely promising for not only fundamental research but also clinical applications, including hepatic arterial infusion. Notably, TMG reportedly reduces nephrotoxicity when cisplatin is administered to mice^28^, and this effect could also be expected with the TMG aq.

In conclusion, the 50 wt% TMG aq. dissolved poorly water-soluble cisplatin. The solubility in 50 wt% TMG aq. was 1.7 wt%, which was 11 times that observed in water. Although the anticancer effect of cisplatin was abolished in DMSO, it was retained in the 50 wt% TMG aq. Furthermore, ^195^Pt NMR revealed that a portion of Pt(NH_3_)_2_Cl_2_ was converted into [Pt(NH_3_)_2_Cl(H_2_O)]^+^ in the 50 wt% TMG aq. On the other hand, the addition of NaCl induced ligand exchange from [Pt(NH_3_)_2_Cl(H_2_O)]^+^ to Pt(NH_3_)_2_Cl_2_. As the medium used in this study contained NaCl, this ligand re-exchange was assumed to occur in the presence of TMG. Moreover, the 50 wt% TMG aq. containing 10 mM cisplatin was freeze-dried. The resulting powder was rapidly solubilised at room temperature; this has not been achieved with conventional cisplatin powders.

## Supplementary Information

Supplementary Information
