# Supplementary material for: High loading of trimethylglycine promotes aqueous solubility of poorly water-soluble cisplatin
Source: Sci Rep. 2021 May 7;11:9770. doi: 10.1038/s41598-021-89144-0 (PMC8105311; doi:10.1038/s41598-021-89144-0)
Supplement: Supplementary file 1 — Supplementary Information [file 41598_2021_89144_MOESM1_ESM.docx]

**Supporting information**

**High loading of trimethylglycine promotes aqueous solubility of poorly water-soluble cisplatin**

Riki Kadokawa, Tetsuo Fujie, Gyanendra Sharma, Kojiro Ishibashi, Kazuaki Ninomiya, Kenji Takahashi, Eishu Hirata,* Kosuke Kuroda*

**Material and Methods**

**Materials**

TMG (betaine anhydrous) and L-carnitine were purchased from Tokyo Chemical Industry Co., Ltd. and used as received. Cisplatin (for pharmacology research) was purchased from Fujiﬁlm Wako Pure Chemical Corporation.

**Solubility of cisplatin in TMG and L-carnitine aqs.**

Cisplatin (3 mg) was added to 300 mg of 50% (w/v) TMG and L-carnitine aqs. by stirring for over 10 h at room temperature. Dissolution of cisplatin was checked by using an invert optical microscope (ECLIPSE Ts2, Nikon Corporation). When it was solubilised, we continued adding cisplatin in 0.1 wt% increments until it was no longer solubilised within 1 h to confirm the solubility.

**Theoretical Basicity**

Lamarche and Platts have developed equation 1 and 2 which can accurately predict basicity of organic solvents using DFT calculation^1^. Among the different computed parameters, hydrogen bond Gibbs free energy (ΔG°) has been found to show best correlation with basicity. Similar method was used to obtain the basicity of TMG, and ΔG° for the most stable 1:1 complexes of hydrogen fluoride and TMG was calculated.

$pK_{HB}=0.481\left( 0.051 \right)+0.080\left( 0.004 \right)\cdot{\Delta G}^{^{\circ}}$ (eq. 1)

$\beta_{2}^{H}=\left( pK_{HB}+1.1 \right)/{4.636}$ (eq. 2)

A complex of TMG and hydrogen fluoride were modelled in four different orientation in gaseous phase at 298.15 K temperature and 1 atm pressure. The modelled molecules were then optimised using Gaussian 09 programme, B3LYP method, and 6-311++G(d,p) basis set^2-4^. The TMG structure was optimised without restrictions, and the obtained geometries were confirmed as minima due to the lack of imaginary frequency analysis. The most stable complex orientation was obtained from four orientations and was used to calculate Gibbs free energy of the hydrogen bond formation. The Gibbs free energy of the hydrogen bond formation is defined as the difference between the Gibbs free energy (corrected by the zero point energy) of the complex (TMG-hydrogen fluoride) and the tolal Gibbs free energy of the pure components (${\Delta G}_{TMG}+{\Delta G}_{HF}$).

The gas-phase hydrogen bond formation Gibbs free energy of the TMG-HF pair was calculated from equation 1, according to Turner *et al*. ^5^.

$\Delta G\left( \mathrm{kJ}\mathrm{mol}^{-1} \right)=2625.5\left[ {\Delta G}_{complex}\left( a.u \right)-\left( {\Delta G}_{TMG}\left( a.u \right)+{\Delta G}_{HF}\left( a.u \right) \right) \right]$ (eq. 3)

The basis set superposition error by the Boys Bernardi counterpoise technique was used to correct Gibbs free energies during the calculation.^6^

**cells**

MDA-MB-231 human breast cancer cell was kindly gifted from Prof. Erik Sahai (The Francis-Crick Institute, UK) and described previously^7^. The cells were grown and maintained as monolayer cultures in 5% CO_2_ and humidified atmosphere at 37 °C, using Dulbecco's modified Eagle medium (high glucose with L-glutamine and phenol red, Fujiﬁlm Wako Pure Chemical Corporation), 10 vol% fetal bovine serum (Sigma-Aldrich Co., Llc.), and penicillin–streptomycin solution (×100; Fujiﬁlm Wako Pure Chemical Corporation). The cells were subcultured every 3–5 days by using trypsin solution (0.5w/v% trypsin—5.3mmol/L EDTA·4Na solution without phenol red (×10), Fujiﬁlm Wako Pure Chemical Corporation).

**Anticancer efficiency of cisplatin**

MDA-MB-231 cells (5,000 cells/well) were seeded to each well of a 96-well plate and then pre-cultured for 24 h. Cisplatin solutions (10 mM) were prepared by using 50 wt% TMG and L-carnitine aqs. or 100% DMSO solutions and left for more than one day. The solutions were added to the media to be 100, 10, 1, and 0.1 μM. The cells were cultured for 72 h and the cell viability was investigated with CellTiter 96^®^ Aqueous One Solution.

**^195^Pt NMR spectrometry and mass spectrometry**

Cisplatin (1 wt%) was added 50 wt% TMG aq. by stirring for 1 h and then was measured by using ^195^Pt NMR (ECA 600; JEOL Ltd.) with scan numbers of 22,000–27,000. After leaving it at room temperature, the resulting solution was measured at Day 5 and Day 10. We used co-axial tubes and K_2_[PtCl_4_] in D_2_O (for adjusting shim) was in the inner tube.

To check re-exchange of ligand of [Pt(NH_3_)_2_Cl(H_2_O)]^+^, 1 wt% cisplatin (10 mg) dissolved in 50 wt% TMG aq. (1 mL) was prepared and left for one day. The resulting solution was measured by ^195^Pt NMR (Day 1; 25,000 scans). NaCl (80 mg) was added and stirred. The solution was left and measured (Day2–4; 25,000 scans). In the case of DMSO aq., the concentration of DMSO uesd was adjusted to be equimolar to 50 wt% TMG aq.

Mass spectrometry was conducted with fast atom bombardment and glycerol matrix was used. Cisplatin (10 mg) was added in water (1 mL). The resulting mixture was filtered and measured.

**Preparation of cisplatin/zwitterion powder**

The platinating agent (10 mM) was added to the 50 wt% TMG aq. (1 mL) and solubilized by stirring for 1 h. The resulting solution was left for more than one day. The resulting solution was freeze-dried and a pale yellow powder was obtained. To investigate the anticancer effect, the powder was added to the ultrapure water to regenerate 10 mM of cisplatin in 50 wt% TMG aq. and subjected to the cell viability assay as mentioned above.

**Figures**


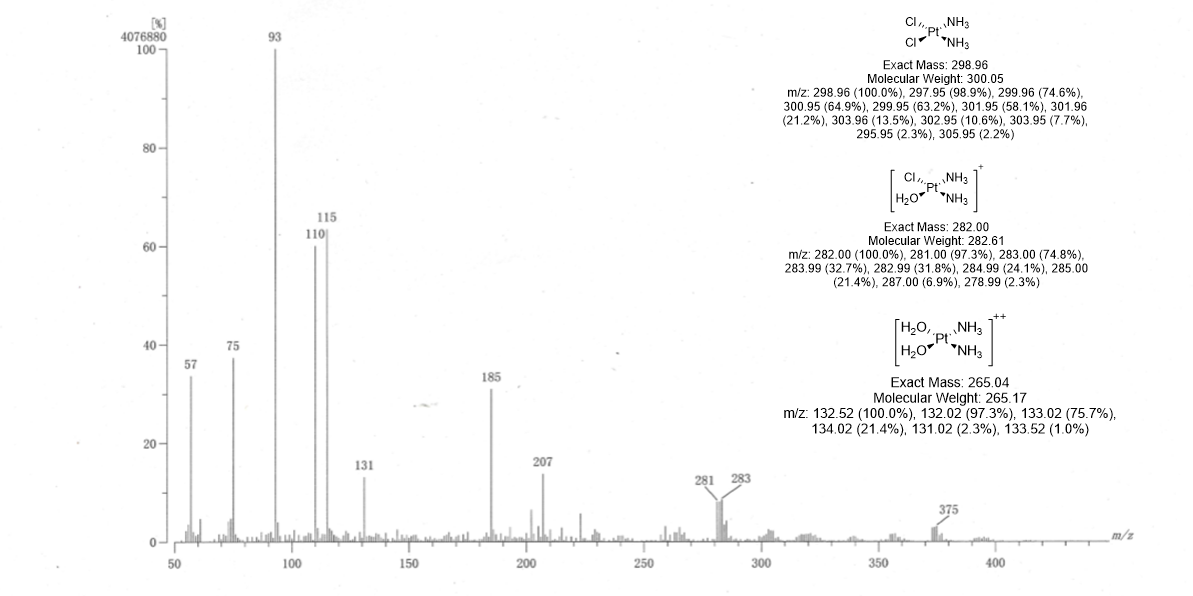


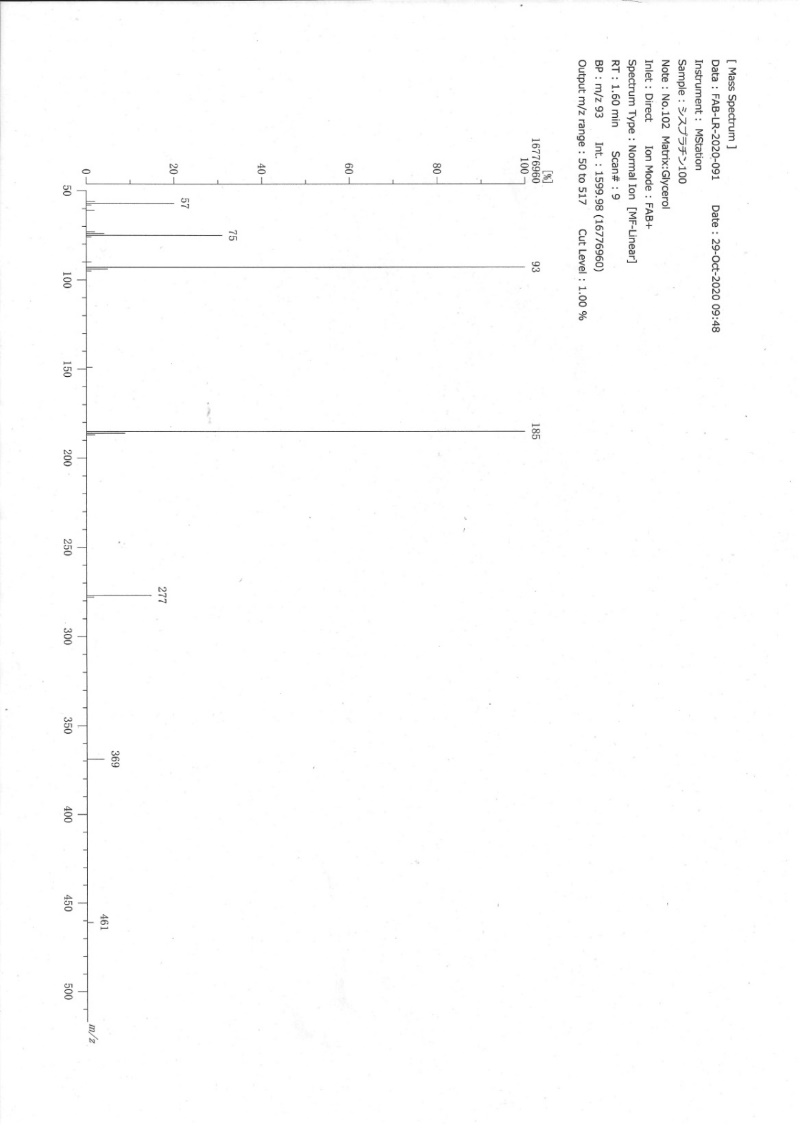


Fig. S1 Mass spectra of cisplatin dissolved in water (top) and glycerol as a matrix (bottom).


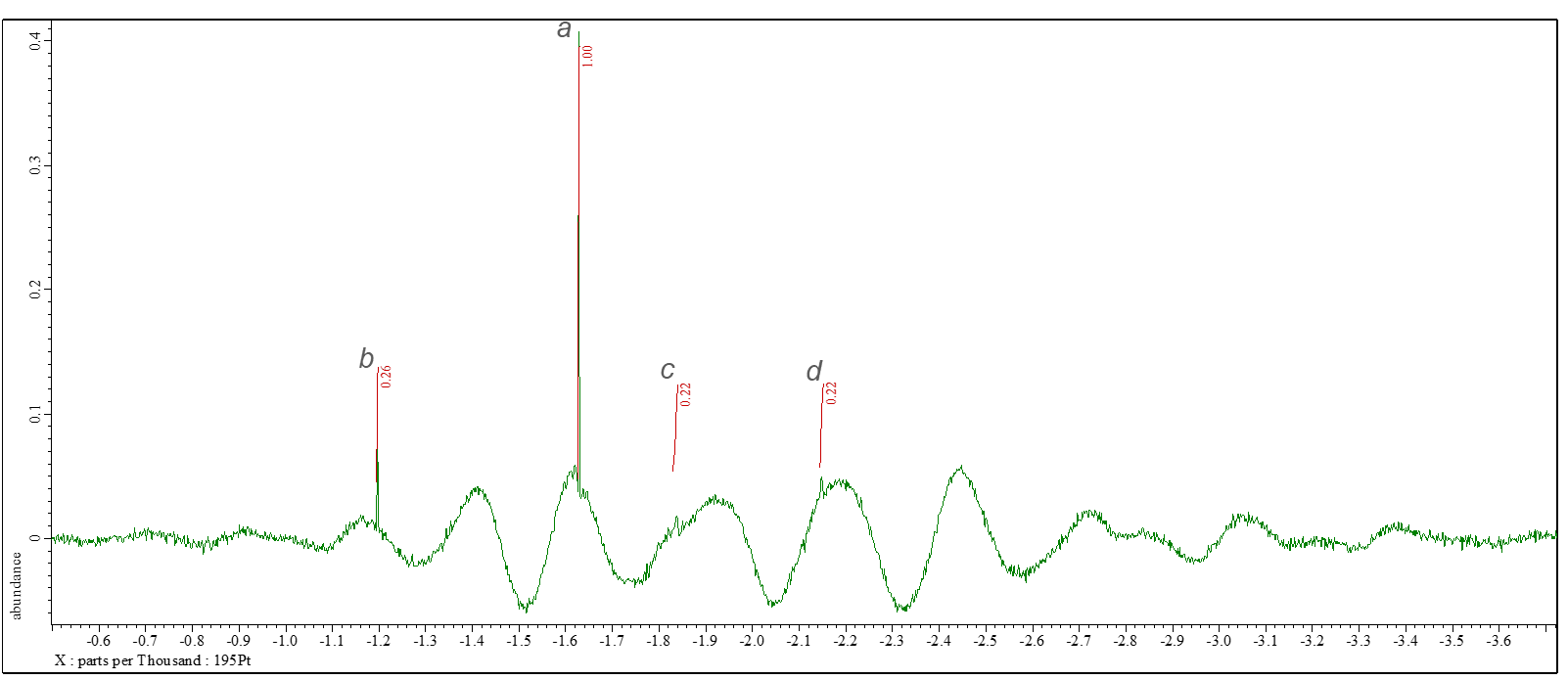


Fig. S2 ^195^Pt NMR spectrum of cisplatin dissolved in water.

*^a^*K_2_[PtCl_4_]; *^b^*K_2_[PtCl_3_(D_2_O)]; *^c^*[Pt(NH_3_)_2_Cl(H_2_O)]^+^; *^d^*[Pt(NH_3_)_2_Cl_2_]


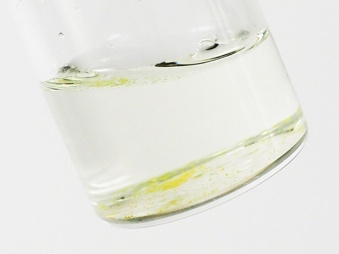

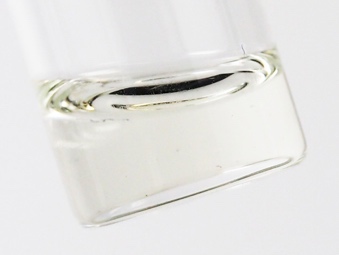


Fig. S3 Megascopic view of the cisplatin (10 mM) added to 50 wt% TMG aq. (left) and the cisplatin/TMG powder added to water (right) after 1 min stirring. The yellow precipitates are undissolved cisplatin.

**References**

1 Lamarche, O. & Platts, J. A. Theoretical Prediction of the Hydrogen-Bond Basicity pKHB. *Chem. Eur. J.* **8**, 457-466 (2002).

2 Frisch, M. J. *et al.* Vol. Gaussian 09 (Gaussian, Inc., Wallingford CT, Gaussian, Inc., Wallingford CT, 2009).

3 Becke, A. D. Density‐functional thermochemistry. III. The role of exact exchange. *J. Chem. Phys.* **98**, 5648-5652 (1993).

4 Lee, C., Yang, W. & Parr, R. G. Development of the Colle-Salvetti correlation-energy formula into a functional of the electron density. *Phys. Rev. B* **37**, 785-789 (1988).

5 Turner, E. A., Pye, C. C. & Singer, R. D. Use of ab Initio Calculations toward the Rational Design of Room Temperature Ionic Liquids. *J. Phys. Chem. A* **107**, 2277-2288 (2003).

6 Meng, Z., Dölle, A. & Robert C. W. Gas phase model of an ionic liquid: semi-empirical and ab initio bonding and molecular structure. *J. Mol. Struct.*–*Theochem* **585**, 119-128 (2002).

7 Hirata, E. *et al.* The Brain Microenvironment Induces DNMT1 Suppression and Indolence of Metastatic Cancer Cells. *Iscience* **23**, 101480 (2020).
